# A scoping review of geographic information systems in maternal health

**DOI:** 10.1016/j.ijgo.2015.11.022

**Published:** 2016-07

**Authors:** Prestige T. Makanga, Nadine Schuurman, Peter von Dadelszen, Tabassum Firoz

**Affiliations:** a Health Geography Research Group, Geography Department, Simon Fraser University, Burnaby, BC, Canada; b Department of Surveying and Geomatics, Midlands State University, Gweru, Zimbabwe; c Department of Obstetrics and Gynaecology, Cardiovascular Sciences Research Centre, St George's, University of London, London, UK; d Department of Medicine, University of British Columbia, New Westminster, BC, Canada

**Keywords:** Geographic information systems, Global health, Health policy, Health services, Maternal health, Spatial access, Spatial epidemiology

## Abstract

**Background:**

Geographic information systems (GIS) are increasingly recognized tools in maternal health.

**Objectives:**

To evaluate the use of GIS in maternal health and to identify knowledge gaps and opportunities.

**Search strategy:**

Keywords broadly related to maternal health and GIS were used to search for academic articles and gray literature.

**Selection criteria:**

Reviewed articles focused on maternal health, with GIS used as part of the methods.

**Data collection and analysis:**

Peer reviewed articles (n = 40) and gray literature sources (n = 30) were reviewed.

**Main results:**

Two main themes emerged: modeling access to maternal services and identifying risks associated with maternal outcomes. Knowledge gaps included a need to rethink spatial access to maternal care in low- and middle-income settings, and a need for more explicit use of GIS to account for the geographical variation in the effect of risk factors on adverse maternal outcomes. Limited evidence existed to suggest that use of GIS had influenced maternal health policy. Instead, application of GIS to maternal health was largely influenced by policy priorities in global maternal health.

**Conclusions:**

Investigation of the role of GIS in contributing to future policy directions is warranted, particularly for elucidating determinants of global maternal health.

## 1 Introduction

Worldwide, at least one woman dies from the complications of pregnancy and delivery every 2 min [1]. For every woman who dies in childbirth, at least 20 more experience long-term life-altering health problems [2]. Furthermore, 99% of such deaths and complications occur in low- and middle-income countries (LMICs), particularly Sub-Saharan Africa and South Asia [3]. Most of these deaths are avoidable because they result from modifiable factors—e.g. prompt recognition of illness, access to transport, and appropriate treatment—that could be addressed through targeted interventions. Maternal outcomes are also influenced by the broad contexts within which individual women live (the social determinants of health); consequently, it is becoming widely accepted that taking action on social factors is an important aspect to improving population health on a global scale [4].

Geographic information systems (GIS) are decision support systems that involve the integration of location-referenced data in a problem-solving environment [5]. The potential application of GIS to health is gaining recognition [6]. Their potential for elucidating risk factors for adverse maternal events, as well as the relationship between access to care and maternal outcomes, has become increasingly apparent. GIS has the ability to integrate data on health-related social and environmental risk factors and thus explain variations in maternal outcomes. This capacity to link the social and environmental risk factors to disease outcomes is consistent with the call to reduce global ill health, including adverse maternal outcomes, through action on social determinants [4].

The present scoping review aimed to investigate what is already known about the use of GIS in maternal health research and practice in both LMICs and high-income countries (HICs).

## 2 Methods

The scoping review method was selected for the present study because it facilitates identification of knowledge gaps and opportunities that exist regarding an emerging subject of interest [7]. A literature review on mapping technologies and methods used within the broad theme of maternal and neonatal health was published in 2015 [8]. Therefore, the focus of the present review was specifically on the use of GIS in maternal health.

The design of the present scoping review was guided by the York method developed by Arksey and O'Malley [7]. The design comprised a five-step process that involved: identification of the questions to be addressed; identification of the relevant literature sources; selection of literature sources to be included in the present review; recording key themes emerging from the literature; and collation, summary, and reporting of the results.

A search was undertaken to identify relevant peer-reviewed articles and gray literature published up to July 31, 2013. No language restrictions were imposed. LMICs were identified using the World Bank classification [9]. The Medline, GeoBase, and Web of Science databases were searched to identify peer-reviewed articles using the terms shown in Box 1. A Google search was performed using the terms “GIS” and “maternal health” to identify relevant gray literature, which included unpublished conference papers and abstracts, descriptions of maternal health programs and initiatives, government websites, books, popular media, and videos. The websites of key organizations (mHealth Alliance, WHO, US Agency for International Development, and United Nations Population Fund) were also searched.

The authors met on separate occasions to review the abstracts and full papers to determine the final set of papers that met the criteria for the review. Articles were included in the present review if they focused on maternal health (prepartum, peripartum, or postpartum) and used GIS in the analysis. Articles that focused on the effect of pregnancy related exposures on neonatal and perinatal outcomes were excluded. Data on the study setting and the key applications of GIS described in each article were recorded and organized into different themes in Microsoft Excel 2010 (Microsoft Corporation, Redmond, WA, USA). Information obtained included the place where the research was conducted (e.g. LMIC/HIC, rural/urban), the nature of the study (e.g. epidemiology, spatial epidemiology, health services), and the type of analysis techniques used (e.g. spatial analysis, statistical analysis).

## 3 Results

### 3.1 Search results

**Fig. 1:**
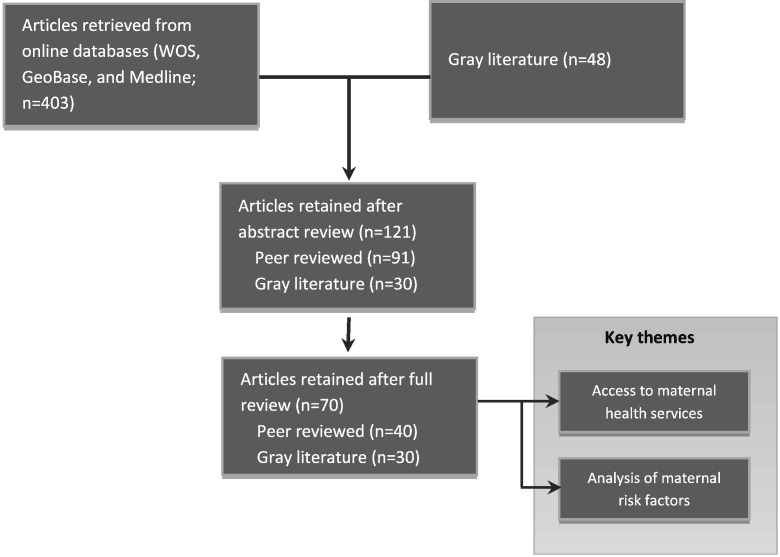
Search results and key themes that emerged from the review. Abbreviation: WOS, Web of Science.

As shown in Fig. 1, the literature search and subsequent review identified 40 peer-reviewed articles and 30 gray literature sources that met the inclusion criteria. Two broad research themes were identified from the selected sources: assessing geographic access to maternal health services, and analyzing risk factors and their associations with maternal outcomes. Articles that covered both of these categories used maps to describe the geographic trends in maternal outcomes, including mortality.

### 3.2 Access to maternal health facilities

The bulk of the published literature regarding the use of GIS in maternal health focused on potential geographic access to care on the basis of the spatial distribution of health facilities [10], [11]. Some articles focused on the use of GIS to describe uptake of maternal services depending on proximity to health facilities [12], [13]. Most papers explored potential spatial access to primary levels of care, including prenatal visits [14]. Few articles covered access to tertiary level care, including facilities with the capacity to deliver emergency obstetric care. In terms of scale, most studies described the spatial patterns for access to maternal care at the national or provincial level [15], [16], with less emphasis placed on community-level trends [17].

Travel distance and time to the health facility based on the road network were the main means for quantifying potential spatial access to maternal care services, particularly among HICs where road network data were readily available [15], [18]. Nonetheless, a large number of studies conducted in HICs used Euclidean (“as the crow flies”) distances to estimate potential spatial access to maternal care. Among LMICs, travel distances based on road network algorithms in GIS were also used to model potential access to maternal care, although in almost all the studies identified, the researchers had to create the road network data in GIS before conducting any analysis, making the research process both time-consuming and expensive [10], [16].

Owing to the unavailability of comprehensive street data among LMICs, some studies used friction surfaces for modeling travel time [19]. This approach is used to model the easiest—and therefore most likely—pathway between communities and health facilities, depending on the travel obstacles that an individual must contend with. Publicly available digital elevation models and data on other potential travel barriers (e.g. bodies of water or land use) were exploited to determine the easiest route to the heath facility and so estimate the travel time. Demographic data were used similarly in LMICs and HICs to align potential spatial access with modes of transport available to populations [17]. For example, Gething et al. [10] used data from populations of reproductive-age women and the transport options available to them to identify subgroups of women expected to need to access maternal care and the time required for them to reach a health facility depending on the mode of transport.

Road classifications and speed limits were used to calibrate the models of potential spatial access to maternal health services. In some instances, clinical records with information on uptake of maternal health services were used to validate the predictive accuracy of spatial accessibility models [20]. Maternal mortality rates in different geographic regions within countries were used to assess the impact of poor access to maternal care on maternal outcomes [21]. None of the reviewed studies in either LMICs or HICs calibrated spatial accessibility models on the basis of measured travel times. Compared with estimated travel times, this approach would have provided a more realistic picture of access to care and matched the realities of the travel experience. The use of GIS in modeling access to maternal care includes assessing the geographic distribution of health facilities as well as modeling the impact of modifying the geographic distribution of health facilities on extending the reach of maternal health services [16].

Some studies used GIS to map the availability of interventions that aimed to improve maternal outcomes. For example, identifying areas with an unmet obstetric need on the basis of standards of care delivery prespecified by WHO [22], [23]. Demographic data were used to quantify the potential need for obstetric intervention among populations, which was then compared with the geographic distribution of health facilities and their capacity to deliver both non-urgent and urgent maternal care [22], [23].

### 3.3 Assessing risk factors for poor maternal outcomes

Spatial epidemiology is defined as the study of spatial variation in disease risk or incidence [24]. This concept is important for advancing the assessment of risk factors for maternal ill-health and utilization of maternal health services [25]. Some risk factors described in the literature fell broadly within the spectrum of social determinants of health and formed the basis for exploring non-biomedical features that characterize the complex pathways leading to poor maternal outcomes. Examples from the literature included risk factors linked directly to characteristics of the physical environment where the pregnant woman lives, including pollution [26] and natural disasters [27], and other risks related to the woman's sociocultural environment, including ethnic origin, education, and poverty [25], [28].

Spatial interpolation is the estimation of values in different locations (e.g. atmospheric concentrations of nitrogen dioxide) on the basis of the measured values at other locations. This technique has been used to model the spread of environmental risks posed by exposure to pollutants during pregnancy [26], [29]. Sociocultural risk factors have been quantified through the use of statistical indicators, such as deprivation, which are usually derived from census data and modeled for populations [30].

The nature and spatial distribution of risk factors for maternal ill-health were generally modeled with either adverse maternal outcomes or a maternal services utilization indicator as dependent variables [25]. The use of geographically explicit methods for modeling the effect of risk factors on maternal outcomes was minimal. Geographically explicit methods include geostatistical techniques and statistical modeling that assumes that statistical associations are affected by geography and therefore not necessarily constant across space. These methods extend beyond simply using GIS to calculate geographic variables, such as travel times and community deprivation scores. Most studies that introduced geographic variables as risk factors into analyses used non-spatial statistical approaches, including odds ratios, least-squares regression, and multilevel models, with the geographic data serving as one of many explanatory variables [31], [32].

## 4 Discussion

The present scoping review found that evaluating access to maternal health services constituted the main use of GIS in maternal health. This finding was not surprising given that increased access to skilled birth attendants through a formal healthcare system is a global priority for improving maternal health [33]. Nonetheless, new approaches must be explored when modeling access to maternal services in LMICs. Most models for accessibility have been developed and tested in HICs; however, 99% of the adverse maternal outcomes occur among LMICs, particularly in rural areas [3], [16]. Many of the existing spatial accessibility models cannot be readily replicated in these highly burdened settings.

The present review identified several knowledge gaps and questions that must be addressed in future work. First, geographic datasets on road infrastructure were scarce among LMICs. Spatial accessibility modeling will therefore require creation of the requisite road network data as a first step [16], a process that often seems to be overlooked by researchers, particularly those from HICs who are conducting research or interventions in LMICs. New protocols are, therefore, required to guide the creation of road network data in resource-limited settings to support mapping of geographic access to maternal care. Second, maternal deaths among LMICs tend to rise during the wet season as a result of reduced access to maternal care owing to precipitation-induced damage to the poor road infrastructure that characterizes many rural areas [34]. The static measures for access to maternal services that currently dominate the literature are, therefore, an inadequate means for quantifying its seasonal variation. The lack of dynamic measures of access to care is a key knowledge gap, suggesting a need for new methods to quantify spatio–temporal access to maternal care that consider the seasonal impact of weather events. Third, community health workers are increasingly being recognized as agents of official healthcare delivery among rural communities in Africa and South Asia [33]. Consequently, models that assess spatial accessibility to maternal care by measuring distance from health facilities, without taking into account how community health workers extend the reach to geographically isolated areas, fail to provide an accurate picture of access to care. Finally, 90% of all armed conflicts since the Second World War have occurred in LMICs, with maternal deaths being disproportionately high in these conflict zones [35], [36]. An important area to address in spatial accessibility modeling is how best to evaluate the impact of conflict on access to maternal health care.

Although GIS are widely used to assess potential spatial access to maternal care, there is a lack of published data evaluating geographic patterns in the association between access to care and maternal outcomes. In most studies, spatial accessibility scores simply serve as input to statistical analyses, together with other variables that are usually non-spatial [12], [19]. Geography thus remains at the periphery of analysis in maternal health research [13]. The use of geographically explicit techniques that explore the spatial structure of associations has been minimal but is receiving more attention from researchers. For example, geographically weighted regression has been used to investigate geographic variation in the association between having medical insurance and access to prenatal health services in the USA [37]. Other examples of tools potentially useful for modeling maternal health risk include land-use regression [26], for modeling spread of pollutants and how these relate to adverse maternal health events. Spatial lag regression [25] also assumes that risk factors in one location are affected by other factors in nearby locations. These approaches might offer insight into the influence of socioeconomic determinants on maternal health. The use of GIS in this way introduces a new geographic dimension to statistical processes and better elucidates the spatial variation in associations with poor maternal outcomes than would conventional statistical techniques.

Although the use of such methods is still novel, the growing “value add” of introducing a geographical perspective to epidemiological research related to maternal health is twofold. First, these approaches might explain the association of risk factors with adverse maternal outcomes, and promote targeted interventions, by highlighting the place-specific patterns that substantially influence adverse maternal outcomes. Conventional statistical methods attempt to collapse patterns in a dataset into a single estimate that best describes the trend in the data (e.g. *R*^2^ or β coefficient); however, the evidence from geographically enabled statistical techniques suggests that parameter values are not always constant across space [37]. Second, geographically enabled statistical techniques tend to improve model efficiency and predictive power [38], largely owing to their increased ability to organize data and fit models to the data on the basis of geography. However, this ability limits the generalization of spatial models beyond the populations where the data were collected, a key limitation given that generalization is an important marker for the utility of health findings. Consequently, although these value additions could increase assimilation of spatially explicit analysis techniques in maternal health research, it remains unknown whether the increased specificity of geographically enabled models is more important than the ability to generalize the results.

To date, the nature of how GIS have been applied to maternal health research and programs for intervention has largely been driven by trends in global health policy concerning maternal health in general. This situation is expected because health GIS comprise an applied discipline and trends in health would obviously determine how GIS are applied. The use of GIS in maternal health research is similar to how this technique is used to evaluate the impact of maternal health programs and mapping maternal outcomes. Reasonable levels of collaboration between academia and the health sector seem to have enabled transfer and refinement of GIS applications.

**Fig. 2:**
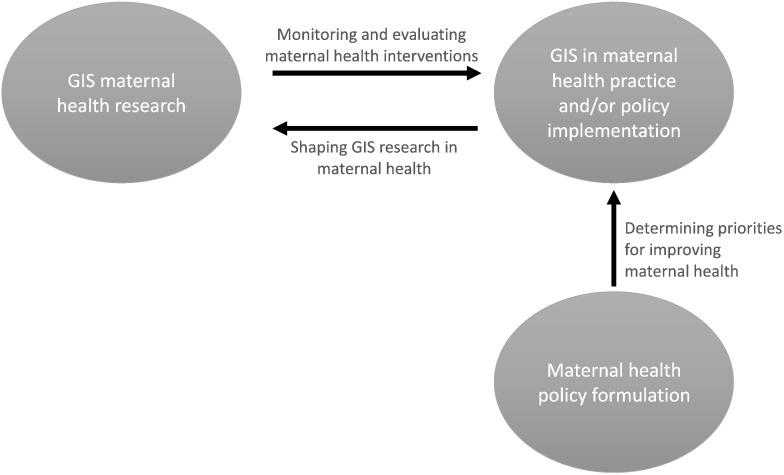
GIS in maternal health and the links to policy formulation and implementation. Abbreviation: GIS, geographic information systems.

The GIS approach has the potential to aid evidence-informed policy formulation because it provides proof for the role of access to care in producing good or bad maternal health outcomes, as well as the means to measure population-based characteristics and how they relate geographically [39]. Nevertheless, the present review found no evidence to suggest that maternal health policy was being influenced by new knowledge emerging from the geographical sciences as they are applied to maternal health. Instead, the application of GIS to maternal health was influenced by policy priorities in global maternal health (Fig. 2) [40]. Clearly, there is potential for GIS to generate further evidence for action to improve maternal health and deliver targeted interventions. Such data are essential, particularly in resource-constrained settings where the burden of adverse maternal outcomes is high and resource allocation must be prioritized.

Efforts to reduce the global burden of maternal ill-health have been driven predominantly by clinical interventions; therefore, GIS have exerted minimal influence on the data. The reason why GIS have remained at the periphery of maternal health policy is that the technology is largely used to evaluate policy implementation, usually on the basis of predetermined indicators, such as access to maternal health services. Increased recognition of the need to promote health through action on social determinants [4] could potentially complement clinical interventions. Examples of social determinants that have been associated with adverse maternal outcomes include maternal education, socioeconomic status, literacy, marital status, and religion [36], [41]. The use of GIS might aid identification of the spatial patterns of these important determinants and explain how they relate to maternal health, potentially offering an integrated approach with appreciable links across sectors, socioeconomic background, and the environment.

In conclusion, the present review has revealed the emergence of GIS in maternal health research and constraints on their implementation. An increased level of sophistication has been observed among GIS methods applied to maternal health; however, their uptake and contribution to policy remains limited. The main focus in the use of GIS has been to develop and improve spatial techniques for evaluating maternal health interventions, particularly access to maternal care. Describing spatial patterns in the burden of maternal ill-health and how these patterns relate to risk factors are also key applications of GIS to maternal health. For example, GIS is used to assess exposure to pollutants among pregnant women during the prenatal period, although the effect of these exposures on neonatal health (rather than maternal health) is in the focus of most published studies.

A number of challenges hamper the use of GIS in LMICs, including the inadequacy of key GIS methods in these settings. The full potential of GIS is also not realized in LMICs owing to inadequacies of spatial data infrastructures to fully support GIS processes in their current form. Approaches developed to assess maternal health in HICs cannot be used in low-resource settings without adaptation to the local contexts. Currently, GIS are being used to evaluate the impact of policy in improving maternal health, with much less done to aid policy formulation related to improving maternal health. There is potential for the exploration of the role of GIS in contributing to new policy directions, particularly in elucidating the role of social determinants in global maternal health.

## Box 1 Sample of search terms used to identify relevant peer-reviewed articles.

**Geographic information systems**

Geography; Mapping; Geographic information systems; Geographic information science; Geographic analysis; Location; Place; Spatial analysis; Spatial epidemiology; Health geography; Medical geography.

**Maternal health**

Health; Maternal death; Maternal mortality; Adverse maternal outcomes; Antenatal; Perinatal; Prenatal; Epidemiology; Referral systems; Indicators; Referral chain.

**Low- and middle-income countries**

Developing countries; Sub-Saharan; South Asia; Africa; Asia; Angola; Burundi; Democratic Republic of Congo; Rwanda; São Tomé and Príncipe; Cameroon

Alt-text: Box 1
