# Corrigendum to “A scoping review of geographic information systems in maternal health” [Int J Gynecol Obstet 134(2016) 13–17]

**DOI:** 10.1016/j.ijgo.2016.09.007

**Published:** 2016-09-28

**Authors:** Prestige T. Makanga, Nadine Schuurman, Peter von Dadelszen, Tabassum Firoz

**Affiliations:** a Health Geography Research Group, Geography Department, Simon Fraser University, Burnaby, BC, Canada; b Department of Surveying and Geomatics, Midlands State University, Gweru, Zimbabwe; c Department of Obstetrics and Gynecology, Cardiovascular Sciences Research Centre, St George’s University of London, London, UK; d Department of Medicine, University of British Columbia, New Westminster, BC, Canada

The authors regret that the Acknowledgments section should have been as follows: “The present study was funded by the Grand Challenges Canada Stars in Global Health program, a Bill & Melinda Gates Foundation grant as part of the Pre-eclampsia Eclampsia Monitoring Prevention and Treatment (PreEMPT) initiative, and the Canadian Institute of Health Research grant KPE-124730.”

